# Is there dietary macronutrient malabsorption in children with environmental enteropathy?

**DOI:** 10.1038/s41430-024-01510-z

**Published:** 2024-10-08

**Authors:** Nirupama Shivakumar, Douglas J. Morrison, Shalini G. Hegde, Anura V. Kurpad, Paul Kelly

**Affiliations:** 1 Division of Nutrition, St. John’s Research Institute, St. John’s National Academy of Health Sciences (A Unit of CBCI Society for Medical Education), Bangalore, India; 2 Center for Doctoral Studies, Manipal Academy of Higher Education, Manipal, India; 3 Scottish Universities Environmental Research Centre (SUERC), University of Glasgow, Glasgow, UK; 4 Department of Pediatric Surgery, St. John’s Medical College Hospital, St. John’s National Academy of Health Sciences, Bangalore, India; 5 Department of Physiology, St. John’s Medical College, St. John’s National Academy of Health Sciences, Bangalore, India; 6 Blizard Institute, Barts and The London School of Medicine and Dentistry, Queen Mary University of London, London, UK; 7 Tropical Gastroenterology and Nutrition Group, University of Zambia School of Medicine, Lusaka, Zambia

**Keywords:** Metabolism, Pathogenesis

## Abstract

Assessing the digestive and absorptive capacity of the gastro-intestinal tract (GIT) using minimally- or non-invasive methods, particularly in children, has been difficult owing to the complex physiology and variability in functional measurements. However, measuring GIT function is increasingly important with the emerging relevance of childhood environmental enteropathy (EE) as a mediating factor in linear growth faltering, severe acute malnutrition, poor oral vaccine uptake and impaired cognition. In EE, sub-optimal nutrient digestion and absorption (malabsorption) forms the critical link to the conditions mentioned above. The present narrative review discusses probable mechanisms that can cause malabsorption of macronutrients, along with mechanistic and experimental evidence, in children (if not, in adults) with EE. The strengths and limitations of the human experimental studies are examined in relation to a battery of existing and potential tests that are used to measure malabsorption. From the available studies conducted in children, lactose and fat malabsorption are more likely to occur in EE. Breath tests (non-invasive) measuring carbohydrate (^13^C-starch/sucrose/lactose), fat (^13^C-mixed triglyceride) and dipeptide (benzoyl-L-tyrosyl-L-1-^13^C-alanine) malabsorption with modifications to the existing protocols seem suitable for use in children with EE. Future research should focus on understanding the degree of macronutrient malabsorption using these tests, in different settings, and link them to functional outcomes (such as growth, muscle strength, cognition).

## Introduction

Environmental enteropathy (EE, pathology) or environmental enteric dysfunction (EED, functional consequence) is an acquired subclinical condition affecting the gut, which is thought to result from a complex interaction between poor nutrient intake, heavy intestinal pathogen burden and impaired host intestinal barrier function. It has been implicated as an important factor in growth faltering, poor cognitive development, and sub-optimal vaccine response in children [1]. In support for the role of EE in growth faltering, children from Zambia, Pakistan and Bangladesh classified at baseline as stunted or at risk for stunting and who were non-responsive to nutritional supplementation (2–6 months), showed evidence of EE (partial villous atrophy with/without intraepithelial lymphocytic infiltration) in varying degrees, on duodenal biopsies [2–4]. However, when associations between EE histopathological score and length or height-for age *z*-score (LAZ or HAZ) were explored in children from Pakistan and Bangladesh, the only significant association was between the intra-epithelial lymphocyte density and HAZ (*ß* = −1.1, 95% CI −1.8 to −0.5) in the Pakistani cohort [3]. This lack of association between the degree of EE and growth, suggests that a biopsy read-out of EE may not fully reflect the functional consequences that negatively influence growth, or simply the multi-factorial nature of growth faltering. The key dysfunction postulated in growth faltering, in EE, is sub-optimal digestion/absorption (malabsorption) of nutrients and/or systemic inflammation [1, 5]. Therefore, there is a need first to first establish the nature and degree of malabsorption in children with EE.

Macronutrients are crucial for child growth, given their role as the main source of energy, and protein in particular as a linear growth regulator [6, 7]. Requirements for protein may be increased in the presence of infection and inflammation (as in EE), which can limit its availability for growth [7]. Malabsorption due to EE, can further compromise this nutrient availability. Thus, an understanding of the type and extent of macronutrient malabsorption, in children with EE, is urgently required. The evaluation of EE has been difficult when using only proxy biomarkers that are non- or minimally invasive measures, while histopathological diagnosis on intestinal biopsy is the accepted “gold” standard [8]. The present narrative review, therefore, aims to examine available evidence of malabsorption in EE, preferably when confirmed by biopsy, or by using other proxy measures. The review attempts to answer the following questions with focus on macronutrients; (1) What are the possible mechanisms that impair digestion and/or absorption of carbohydrate, protein, and fat, in EE? (2) Is there experimental evidence for reduced digestion and absorption of these macronutrients in EE? Whether EE-related malabsorption can explain linear growth faltering in children is difficult to establish, as other biological mechanisms (like growth signaling) related to EE but potentially distinct from macronutrient malabsorption can also influence growth [7] and this question is therefore not discussed in this review. The strengths and limitations of the human experimental studies are examined in relation to a battery of tests used to measure digestive and absorptive functions of the gut. Finally, research gaps related to mechanisms and experimental evidence, and directions for future studies using functional measures of the gut are provided.

## Search strategy and selection criteria

References for this review were identified through searches of PubMed and Google Scholar. Search terms were specifically strung together for the questions related to mechanism, evidence, and the tests used to measure macronutrient malabsorption, in children or adults, and animals where necessary. The following search terms were used, “adults” or “children” or “animal”, “absorption” or “digestion” or “malabsorption”, “lipids” or “fat”, “carbohydrates”, “protein” or “amino acids” or “peptides”, “enteropathy” or “environmental enteropathy”, “exocrine pancreatic insufficiency”, “tests” or “breath tests” or “pancreatic tests”, “severe acute malnutrition”, without using a year filter. Boolean operators ‘AND’ and ‘OR’ were included to ensure comprehensiveness. This was complemented with additional searching of cross-references in the eligible articles and “similar articles” on PubMed. Only papers published in English were reviewed. For the question on experimental evidence, studies were eligible if their primary focus was on measuring digestion or absorption of macronutrients, in children or adults with either a biopsy confirmation or by using a proxy measure of EE. The final reference list was generated on the basis of originality and relevance to the broad scope of this review. A narrative approach was adopted as the review dealt with connecting questions, wherein mostly theoretical deductions were made for the section on mechanism that needed supporting literature, followed by evidence, which was further complemented by tests to measure malabsorption.

## How can digestive and absorptive functions of the gut be measured in children?

The tests used for estimating digestive or absorptive capacity of the pancreas or the intestinal brush border membrane (BBM) are described in Table 1. Other relevant articles, which consists of tests that assess clinical pathology of gastric acid secretion, pancreatic exocrine (secretory) function (direct-invasive and non-invasive, and indirect tests), small intestinal malabsorption, and breath tests, along with their interpretation and disadvantages, have been reviewed in the past [9, 10]. Inclusive of the tests mentioned in both the past reviews, Table 1 provides details of up-to date, in current use, established and potential in vivo tests for macronutrient digestion and absorption, with focus on their relevance to children with EE. The table comprises information on the function probed by each test, standardization (against a gold standard) and validation of test protocols, the degree of insufficiency/impairment the test detects, normal values in children, and their disadvantages.

**Table 1:** Tests for assessing macronutrient digestion and absorption.

	Type of method	Test	Function tested	Standardization of protocol and validation against criterion standard	Detection	Age-specific normal values for children	Cost^a^	Disadvantages	Relevance to children with environmental enteropathy	References
Digestion										
Carbohydrate, protein and fat	Direct (invasive)	Endoscopic pancreatic function test (ePFT) or pancreatic stimulation test (PST) using secretin and/or cholecystokinin (CCK)/caerulein as stimulant.	Pancreatic bicarbonate and enzyme secretion (amylase, lipase, trypsin) in the duodenal contents following stimulation.	• Multiple protocols in use, not standardized in children • Accepted as a criterion standard for exocrine pancreatic enzyme output	Mild to severely reduced stimulated pancreatic trypsin, lipase and amylase output.	Not established. Lab specific values for children are provided [50]	High	• No age-specific values • Technically challenging • Invasive, labor-intensive and expensive	Although suited to detect sub-clinical derangement, its use is limited by the disadvantages.	[51]
Digestion and absorption										
Carbohydrate	Indirect (minimally invasive)	Oral tolerance or loading test with a glucose response curve. Sugars used are lactose, sucrose and glucose.	Global read out of intestinal brush border enzyme (hydrolysis) and transporter activity (absorption), and subsequent metabolization of substrates.	• Protocol standardized in children • Validated against mucosal biopsy disaccharidase activity in children (age ranging from 3 months to 14 years) [52]	Not clear	Although not validated, if the blood glucose rise is <30 mg/100 mL after the oral carbohydrate administration, then intolerance is considered likely; increments of <20 mg/100 mL is considered diagnostic of malabsorption.	Low	• High false positives, due to variation introduced by splanchnic extraction, glucose oxidation, and the influence of insulin or other hormones • 3 blood samples required	Has been used in EE (Table 1) but is shown to be of limited value in estimating the digestive/absorptive capacity [53].	[50, 52]
	Indirect (non-invasive)	^13^C-starch breath test plus ^13^C-glucose breath test, conducted on separate days. The ^13^C-glucose breath test is performed to normalize individual test substrate oxidation, called Coefficient of Glucose Oxidation (CGO%).	Global read out of starch digestion, by salivary and pancreatic amylase, followed by absorption and subsequent metabolization of substrates.	• Protocol *not* standardized in children • Validated against criterion standard for impaired pancreatic function (mild to severe EPI), in adults [54]	Severely reduced stimulated pancreatic trypsin, lipase and amylase output	Not established. One study [55] reports median (range) cumulative percentage ^13^C dose recovery (cPDR) in breath of 35% (18–52%), in *n* = 5 normal healthy controls (age range 7–13 years; 3 girls, 2 boys) from Italy.	Medium	In children <2 years, • Previous day diet/breastmilk with a higher abundance of ^13^C, which could be difficult to avoid/control, could dilute the body’s ^13^C pool • Uncontrolled/excess activity, which could lead to a faster washout of the ^13^C in breath • An additional testing day for the glucose reference experiment may be taxing	Can be explored. Needs standardization of protocol for use in children. Improvisation of the test as for lactose breath test (see below) can be considered.	[53, 54]
		^13^C-sucrose breath test (SBT) plus 13C-glucose breath test (separate day) for CGO%.	Global read out of brush border sucrase-isomaltase enzyme activity, absorption, and subsequent metabolization of substrates. Rate limiting step not determined.	• Protocol *not* standardized for children • Validated against mucosal biopsy sucrase activity for detection of low duodenal sucrase activity (values in the range of 0 to 6.5 U/g protein) in children (age range 1–15 years, *n* = 10) [56]	Not clear	Not established. On study reports an average of 146% ± 45.5 mean % CGO, in children (2–15 years, *n* = 10), from Houston, United States [56]	Medium	As mentioned for ^13^C-starch breath test	A modified 4 h protocol of ^13^C-SBT without CGO% is being evaluated in children with a narrow age range of 12–15 months, tested for suspicion of EE [57] Improvisation of the test as for lactose breath test (see below) can be considered.	[55, 56]
		^13^C-lactose breath test (LBT)	Global read out of brush border lactase enzyme activity, absorption, and subsequent metabolization of substrates. The rate limiting step was noted to be glucose oxidation in an adult study. Here, the researchers incorporated an exercise protocol to increase glucose metabolism and shifted the rate limiting step to lactose hydrolysis [58]	• Protocol *not* standardized for children • Validated against mucosal biopsy lactase activity of less than 10 U/g protein, in children (age range 1–19 years, *n* = 27) [59]	Not clear.	Not established. One study report mean (SD) 13C-LBT 4 h cPDR of 21.5% (4.8%), in *n* = 21 healthy schoolchildren (from Netherlands) aged 4–8 years without signs of lactose maldigestion and with a negative lactose H2 breath test [59]	Medium	As mentioned for ^13^C-starch breath test	Modification to the existing protocol considering an age-specific standardization of an exercise/physical activity regime, may prove beneficial in detecting mild-moderate lactase deficiency. Further improvisation of the test can be made by co-administering ^2^H-glucose (internal reference) with ^13^C-lactose, which compensates for interfering factors such as gastric emptying, small intestinal transit time and insulin effects. The ratio ^13^C-glucose/^2^H-glucose concentration in serum, is considered to reflect lactose digestion capacity.	[57, 58]
Protein	Indirect (minimally invasive)	Dual tracer approach: Intrinsically ^2^H-labeled animal or plant indispensable amino acid (IAA) digestibility using differentially labeled free IAA mixture as a reference.	Protein or specific IAA digestion. Whole protein is used as a test substrate for pancreatic or brush border proteases or peptidases.	• Protocol with free IAA mixture as reference not standardized in children • Not validated against a direct method, such as oro-ileal balance in ileostomates, or through naso-ileal intubation, which is considered to be relatively more accurate	Not clear	No data in healthy children from a high socio-economic status.	High	• Labor-intensive • 3 blood samples required	Potential test for use in children with EE, using free IAA mixture as reference. Has been tested in children with suspected EE, using labeled mung bean and spirulina, to determine IAA systemic availability, along with phenylalanine digestibility and absorption.	[44, 59]
	Indirect (non-invasive)	Breath test using ^13^C-egg white protein-L-[1-^13^C]-leucine-substituted eggs or whole egg with Pancake test meal.	Whole protein is used as a test substrate for pancreatic or brush border proteases or peptidases.	• Protocol *not* standardized for children • In adults, logarithmic correlation between cPDR 6 h and stimulated trypsin enzyme output after maximal stimulation with caerulien was 0.88 [60]	Not clear	No studies conducted in children.	High	First 2 points as mentioned for ^13^C-starch breath test	Can be explored. Needs standardization of protocol for use in children.	[60]
		N-benzoyl-L-tyrosyl-p-aminobenzoic acid (BT-PABA): urine excretion or peak serum value.	Synthetic tripeptide that is a substrate for pancreatic chymotrypsin, cleaves the PABA, which is then absorbed, partially conjugated in the liver and excreted in urine.	• Protocol *not* standardized for children • Validated against criterion standard for mild to moderate impairment in pancreatic enzyme secretion, in adults [61]	Severe, less sensitive for mild and moderate reduction in stimulated pancreatic trypsin, lipase and amylase output	A study in children (0–12 years, *n* = 48) from Italy, reports a mean (SD) for urinary PABA excretion of 59.4 (26.7) % and serum peak PABA of 3.7(1.4) ug/mL [62]	High	• 6 h of urine collection, which could be cumbersome in children <3 years • Intact NT-PABA absorption through peptide transporters has not be determined	The test may not be suitable, with poor sensitivity for mild to moderate reduction in pancreatic enzyme output.	[61, 62]
		^13^C-labeled dipeptide: benzoyl- L-tyrosyl-L-1-^13^C-alanine breath test	Substrate for pancreatic carboxypeptidase A, cleaves 13C-alanine, which is then absorbed and metabolized. Alanine absorption is assumed or should be normal, or a separate experiment is performed to test for absorption of 13C-Ala, which is transported by neutral amino acid transporters in the brush border membrane.	• Protocol *not* standardized for children • Not validated against pancreatic carboxypeptidase A activity	Not clear	No studies conducted in children.	High	First 2 points as mentioned for ^13^C-starch breath test • Two experimental days are required if testing for alanine absorption • Peptide absorption through peptide transporters not determined	Can be explored. Needs standardization of protocol for use in children and validation against direct pancreatic stimulation.	[63]
Fat	Indirect (non-invasive)	Quantitative (72-h) fecal fat estimation: Coefficient of fat absorption (CFA)	Global read out of fat digestion and absorption, reflecting Lipolysis (pancreatic lipases), solubilization (bile acids) and intact small intestinal mucosa and/or transporters for absorption. Indirectly reflects pancreatic trypsin and amylase output.	• Protocol standardized in children • Validated against criterion standard to detect ≤10% of stimulated pancreatic trypsin, lipase and amylase output [64] • Considered criterion standard for indirect estimation of fat malabsorption	Severely reduced stimulated pancreatic trypsin, lipase and amylase output	The range of normal values with a nomogram for fecal fat concentration (FFC, %) and fecal fat excretion (FFE, g/d) are reported age-wise for normal growing Poland children (age range 1–24 months, *n* = 160) [65] However, this study did not subject children to a diet with fixed fat content, although fat requirements (as assessed by dietary records) were met.	Low	• A fixed fat diet in children reduces compliance • The effect of poor absorption of fat due to intestinal mucosal damage is not clear	The test may not be suitable, considering poor sensitivity for mild to moderate reduction in pancreatic enzyme output.	[64, 65]
		^13^C-mixed triglycerides (^13^C-MTG)	Lipolysis (pancreatic lipases), solubilization (bile acids) and intact small intestinal mucosa and/or transporters for absorption. The rate-limiting step in the oxidation to ^13^CO_2_ is the hydrolysis of the fatty acids in the 1 and 3 positions of the mixed triglyceride.	• Multiple protocols in use, dose (wide range 4–15 mg/kg), standard meal and duration (4–6 h) *not* standardized for children • Validated against criterion standard, in adults [66]	Moderate to severe. Better detection for severely reduced stimulated pancreatic trypsin, lipase and amylase output	Normal values for Belgium (Leuven) children has been reported by for, full-term infants (1–6 months, *n* = 12), children (3–10 years, *n* = 20), and teenagers (11–17 years, *n* = 20). In children the mean ± SD 6 h cPDR was 32.5 ± 5.3% (range: 24.5–44.1). In teenagers the mean 6 h cPDR was 28.0 ± 5.4% (range: 15.4–34.8) [67].	Medium	First 2 points as mentioned for ^13^C-starch breath test	An abbreviated (4-h) protocol with an increase in fat content of the meal given with the ^13^C-MTG dose can be explored to detect a mild to moderate reduction in lipase secretion.	[66, 67]
		^13^C-hiolein (labeled mixture of triglycerides)	Lipolysis (pancreatic lipases), solubilization (bile acids) and intact small intestinal mucosa and/or transporters for absorption.	• Dose and meal *not* standardized in children • Validated against criterion standard for a reduced duodenal lipase output (between 12,000–65,000 U/30 min), in adults	Severe with lipase output <10% and less sensitive for mild to moderate reduction in pancreatic lipase output.	Not established. No data in children for hiolein.	Medium	First 2 points as mentioned for ^13^C-starch breath test • Long (6–12 h) duration of breath collection	Hiolein is not feasible in children unless the 12 h protocol is abbreviated.	[68]
		^13^C-triolein		• Validated against 72 h fecal CFA, in children (age range 3 months–17 years) with (7–63.5% of fat intake excreted per day) and without steatorrhea	Not clear	The cumulative % dose excretion over 6 h (mean ± SD) for triolein in normal controls (Chicago, US, *n* = 10, age range 2.5–8 years) was 11.3 ± 6.7%, and the peak % dose/h was 5.0 ± 2.0%.	Medium		Triolein can be tested, with dose and duration modification similar to ^13^C-MTG.	[69]
		Fecal elastase-1	Elastase-1 is the proteolytic enzyme secreted by exocrine pancreas (~6% of total pancreatic output) that is normally not degraded during intestinal transit. It reflects the level of pancreatic output and also correlates with the output of other pancreatic enzymes such as lipase, amylase, and trypsin.	• Standard ELISA kits (Enzyme immunoassay using mono- or polyclonal antibodies directed against human pancreatic elastase) are commercially available • Validated against criterion standard, in children (age range of 8–76 months), for detecting low pancreatic enzyme output [70]	Moderate to severely reduced stimulated pancreatic trypsin, lipase and amylase output	In normal growing Poland children (age range 1–24 months, *n* = 160), range of FE-1 was 200 to 1695 μg/g of feces. In the first 3 months FE-1 values were lower than in the second year of life (1–3 months versus 13–24 months), reaching a plateau around the age of 6–10 months [71]	Low	• Luminal inactivation by SIBO not determined	Potential for use as a screening test. FE-1 at values <100 ug/g is likely to be associated with intestinal mucosal inflammation and damage [27]	[27, 70, 71]
		Fasting breath hydrogen concentration (FBHC)	EPI induced change in the intestinal bacterial composition with possible predominance of Clostridium species (hydrogen producing bacteria)	• Protocol *not* standardized in children • Not validated against criterion standard	Not clear	No studies conducted in children.	Low	• Fasting period for children not determined, which may limit its use in children <2 years	May be developed as a screening test. Requires validation in children with EPI. The effect of SIBO needs to be determined.	[72]
Absorption										
Carbohydrate	Direct (invasive)	Perfusion technique: aspirating jejunal contents through a double lumen naso-jejunal tube, at set intervals after administering different types of carbohydrates to calculate the sugar absorption rate (as mmol/hr/cm of jejunal segment)	SI brush border disaccharide hydrolysis and monosaccharide transporters in the selected segment.	• Dose and infusion rate *not* standardized in children • Can be considered as the functional criterion standard for direct estimation of absorption	Not clear	No data in healthy children.	High	• Absorption localized to a short segment • Possible contamination by endogenous secretions • Invasive • Protocol (2 h) needs to be repeated for each sugar separately	May be used to validate indirect non-invasive breath tests, by using labeled substrates to overcome the issue of endogenous contamination and repeated testing.	[52]
	Direct (minimally-invasive)	Dual tracer approach: U-^13^C labeled glucose for oral administration and [6,6-(2)H_2_]glucose for intravenous administration to calculate % of glucose absorption.	SI brush border glucose transport, with first pass splanchnic extraction/metabolism.	• Dose and infusion rate *not* standardized in children	Not clear	Limited data in children	High	• Physically demanding for young children	May be used to validate glucose absorption in comparison to indirect non-invasive breath tests.	[73]
Protein	Direct (invasive)	Perfusion technique (as explained above)	SI brush border amino acid transporters in the selected segment.	• Dose and infusion rate *not* standardized in children	Not clear	No studies conducted in children	High	• Absorption localized to a short segment • Possible contamination by endogenous secretions • Invasive	May be used to validate indirect non-invasive breath tests, by using labeled substrates to overcome the issue of endogenous contamination.	[45]
	Indirect (minimally-invasive)	Dual tracer approach: ^13^C_6_,^15^N-L-allo-isoleucine for oral boluses and ^2^H_10_-L-allo-isoleucine for intravenous administration to calculate allo-isoleucine absorption index (%).	SI brush border neutral amino acid transport. Allo-isoleucine is a fairly inert molecule, with minimal oxidation and is not utilized for protein synthesis.	• Dose and infusion rate *not* standardized in children	Not clear	No studies conducted in children	High	• Physically demanding for young children	May be considered after modification to, route of administration, duration and minimizing number of blood sampling.	[74]

^a^As costs vary depending on price of isotope, number of biological samples analyzed, personnel costs incurred, and analytical costs, they are provided on a relative scale to each other, as low, medium and high.

The following characteristics are preferable in tests that measure intestinal function in young children with EE, (1) non-invasive or minimally invasive, (2) detect sub-clinical or mild to moderate degree of derangement and (3) ensure participant compliance through short duration testing, less frequent biological sampling, acceptable and standardized test meals, and experiment pre-requisites such as short duration of fasting (in children <2 years). Of the tests discussed in Table 1, the direct pancreatic stimulation test could detect mild-moderate deficiency in pancreatic enzyme output but is invasive and is limited by compliance. The carbohydrate, fat and dipeptide breath tests using ^13^C-starch/sucrose/lactose/mixed triglyceride and benzoyl-L-tyrosyl-L-1-^13^C-alanine respectively, meet the criteria of being non-invasive and are relatively easy to perform, with test time varying between 1.5–6 h. A number of these tests, however, lack standardization/validation in the pediatric population, with no clarity on the degree of derangement they detect. Additionally, the normative cut-offs have not been established, and differ from lab to lab. Nevertheless, if these limitations are addressed, there is potential for improvisation of the existing tests for their use in children with EE.

## Macronutrient malabsorption in EE

To understand the impact of EE on macronutrient digestion and assimilation, knowledge of the site, extent and timing of EE is necessary for the following reasons; (1) there is a proximal to distal intestinal gradient of brush-border membrane (BBM) enzymes and nutrient transporters [11], (2) intraluminal proteolytic degradation of pancreatic enzymes can occur during duodenum-ileal transit (lipolytic activity is the most susceptible to inactivation) [12], (3) the influence of ontogeny of pancreatic and BBM enzymes with age, and the influence of endogenous hormones and exogenous (diet, environmental stressors) factors [13, 14] and (4) secondary or functional exocrine pancreatic insufficiency (EPI) can occur due to impaired entero-endocrine pancreatic signaling from the proximal intestine [15]. These mechanisms underscore the spatial and temporal relevance of the highly organized intestinal delivery and digestion of macronutrients in the intestine.

There is an established duodenal-ileal gradient for absorption of glucose, AA, and peptides; AA absorption is greater in the distal small intestine than proximal, which is reverse in case of di- or tripeptides [13, 16]. Sucrase-isomaltase and β-glycosidase activities are higher in proximal jejunum, while glucoamylase is high in proximal ileum. Peptide and long-chain fatty acid transporters show predominance in the duodenum and jejunum [11]. Lipase and proteases lose ~35% of their activity during the duodenum-jejunal transit, with further loss (>40%) in transit to the ileum, as observed in response to infused macronutrients [12]. Lipolytic activity is the most susceptible to inactivation compared with proteolytic and amylase activity, and loss of elastase is minimal. The survival of the pancreatic enzymes depends on the interaction between delivery of nutrients, and the location/rate of absorption of nutrients and bile acids [12]. The BBM disaccharidase lactase-phlorizin hydrolase (LPH) activity, which is present along the entire length of the small intestine at birth shows a gradual decline with cessation of breastfeeding in many but not all ethnic groups, whereas sucrose-isomaltose (SI) activity and fructose absorption increases with introduction of complementary foods [13]. Pancreatic amylase activity that is undetectable at 1 month of life, increases subsequently with the introduction of complementary foods reaching adult levels by 2 years of age [14]. It is noted that the intestinal response to a diet depends on the age of the child, the duration, and any previous exposure [13]. Overall, studies set to determine digestive and absorptive capacity in early life (<2 years of age), should ensure that there are age-matched controls and consider evaluation of pre-experimental dietary patterns.

However, there is little information on the extent or intensity of damage of the small intestine in EE, as multiple biopsy sampling across the entire length of intestine is not feasible nor ethical in children [3]. In Zambian adults at least, EE does not affect the ileum (P Kelly, unpublished observations). Thus far, studies have only been able to characterize the degree of gross and histopathological lesions at selected sites of the intestine, mainly the duodenum and jejunum, showing partially or fully atrophied villi with loss of secretory cell lineages or a higher density of intraepithelial infiltrates [3, 4, 17, 18]. Children with severe acute malnutrition (SAM), are known to suffer from a severe form of EE, showing marked duodenal villous atrophy [19]. In addition, recent evidence from rhesus monkeys (EE model), where disrupted colonic barrier explained growth faltering, suggests the possibility of the colon being affected in children with EE [20]. This opens up new questions (Box 1) requiring further investigation into the extent of EE, considering the potential contribution of large intestinal dysfunction to the body’s nutrient economy, through energy harvesting and adaptation [21, 22]. With the advent of new technologies (such as wireless capsule endoscopy) these questions can be investigated in children with EE [1].

**Fig. 1:**
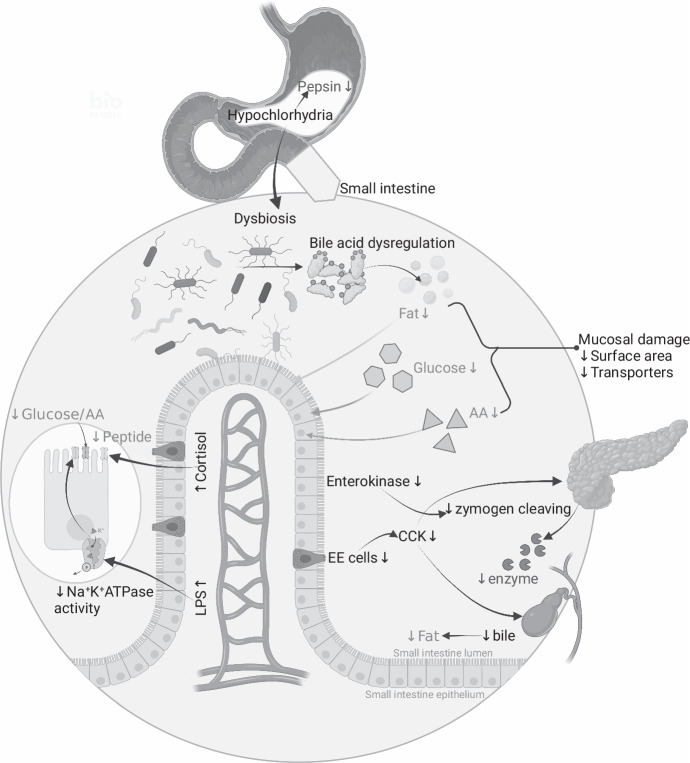
The figure illustrates potential mechanisms that could impair digestion or absorption of macronutrients in environmental enteropathy. Starting from the gastric cavity, hypochlorhydria reduces pepsin activity (may impair digestion) and leads to dysbiosis in the small intestine. Dysbiosis results in bile acid dysmetabolism and reduced fat absorption. Dysbiosis also plays a role in mucosal damage that reduces surface area for absorption, and transporter density. Enterokinase levels may be affected in mucosal damage leading to reduced pancreatic enzyme activity (may impair digestion). Reduced enteroendocrine cell density because of mucosal damage, reduces CCK release that in turn decreases pancreatic enzyme release, which impairs macronutrient digestion. Elevated levels of cortisol or E-coli toxin LPS could also reduce peptide, glucose and AA transport across the brush border membrane. The downward arrow indicates reduction, upward arrow indicates increase in levels. AA amino acids, CCK cholecystokinin, EE enteroendocrine, LPS lipopolysaccharide. Figure created with BioRender.com.

The present section attempts to enlist mechanisms (Fig. 1, theoretical possibility or evidence) that can cause macronutrient malabsorption, in EE, followed by available experimental evidence in children (if not, in adults), and discusses gaps in research with a note on how to investigate them.

Box 1

- Does intestinal damage differ along the small and large intestine?
- Do the functional consequences depend on the involvement of the colon?
- What is the contribution of nutrient competition for the intestinal microbiota or parasites, to macronutrient malabsorption?

### Mechanisms common for all macronutrients

The human intestinal barrier function is dependent on healthy structure and function of the epithelium, mucus layer, secreted antimicrobial factors, and microbiota [23]. Small intestinal bacterial overgrowth (SIBO), an extreme form of dysbiosis, is a well-known entity in EE [4, 24]. Dysbiosis can trigger dysregulated epithelial apoptosis, which could indirectly disrupt the “pore”, “leak” or “unrestricted” paracellular pathways (global barrier loss) [23]. A vicious cycle of hyperimmune response and intestinal inflammation can ensue, and result in perpetuated mucosal damage [23]. This mucosal damage or villous atrophy, as noted in EE, implies a reduced surface area/transporters for nutrient absorption, impaired enterokinase activity (enzyme converting zymogens to their active form) and entero-endocrine-pancreatic signaling leading to EPI and postcibal asynchrony [15, 25]. Overall, these mechanisms could differentially impair digestion and absorption of the macronutrients. Only if EPI is severe enough (trypsin and lipase <10% of the normal output) will it have a significant impact on absorption of protein and fat, reflecting the high functional reserve capacity of the pancreas [26]. Carbohydrate (CHO) digestion is the least affected, as unlike protein or fats it is well compensated by salivary and gastric amylase, with further breakdown (fermentation) by colonic bacteria [15].

### Evidence for EPI

There is evidence of EPI being associated with duodenal enteropathy (of varying etiology), in children [27]. Children showing mild to severe partial villous atrophy or flat mucosa or lymphocytic infiltration of lamina propria on duodenal biopsies had pancreatic insufficiency of varying degree, which was determined using fecal elastase-1 assay [27]. Furthermore, apparently normal Senegalese children (mean age ~1 year) at high risk for EE, were reported to have sub-optimal pancreatic exocrine output, with lower amylase (35% of normal), lipase (6.8% of normal), trypsin and chymotrypsin (~40% of normal) when compared to age-and sex-matched (aged ~1 year) French children, which was then termed “silent pancreatic insufficiency” [28]. The possibility of this relatively low enzyme output causing malabsorption is more likely for fat (with <10% lipase output) than for protein or carbohydrates [15]. A study in adults with severe pancreatic insufficiency (<5% of normal enzyme output) reported a higher (7 times, as energy) ileal cumulative nutrient delivery, that is ~40% of the administered easily digestible low-calorie meal was malabsorbed [29]. In addition to this, there was accelerated gastric and small intestinal transit (2 times) and premature transition from fed to inter-digestive motility pattern compared to normal adults [29].

From these findings, EPI seems to be one of the potential mechanisms, depending on severity, by which EE could cause malabsorption of macronutrients. This suggests the possibility of pancreatic enzyme replacement therapy (PERT) to support digestion, in children with moderate to severe EE, and has been tried before. A reduction in mortality (19% versus 38%) but no difference in weight gain was noted on administration of PERT (containing lipase, amylase and protease), at a dose of 3000U of lipase/kg body weight for 28 days, in children (mean ± SD age of 20 ± 12 months) with SAM, in comparison to those who did not receive therapy [30]. In children (age range 6–30 months) with coeliac disease (similar to EE) with sub-normal pancreatic enzyme output as observed in duodenal aspirates, PERT (first 30 days) has shown a significant percentage increase (9 versus 5%) in weight-for-age, in comparison to a control group on placebo [31].

Mechanisms specific for each nutrient has been described under each macronutrient, below. In EE, these mechanisms may operate individually or synchronously, and an additive effect may substantially reduce nutrient assimilation, which may be buffered by compensatory adaptive mechanisms by the host, mainly in the gut.

#### Carbohydrates

##### Mechanism with supporting evidence

Apart from the general mechanisms described above, a direct inhibitory effect of the E-coli toxin lipopolysaccharide (LPS) has been observed on D-glucose transport, in jejunal mucosa of rabbits [32]. LPS seems to alter receptor affinity at the BBM and the basolateral membrane Na^+^, K^+^-ATPase activity [32]. The smallest dose (range used 3 µg/mL to 3 × 10^−5^ µg/mL) of LPS used in this experiment that had an inhibitory effect on the transporters was 3 × 10^−5^ µg/mL. The plasma LPS concentration in children (2–17 months at recruitment) with non-responsive (4–6 months of nutritional supplementation) stunting and EE has been noted to be in the range of 3–6 × 10^−2^ µg/mL^2^. At this concentration, there could be intestinal mucosal or serosal inhibition of nutrient transporters in these children, as observed in the rabbit model. A direct evaluation of this mechanism is difficult in-vivo, especially in children, however, breath tests using carbohydrate substrates (Table 1) can be used to test malabsorption in children with EE and associations can be drawn to circulating LPS concentrations.

##### Evidence for carbohydrate malabsorption

Table 2 summarizes studies in *children* with features of EE on small intestinal biopsy samples, and who have undergone measurements for macronutrient malabsorption. Studies in children, with suspected EE (lacking biopsy confirmation), in whom digestion or absorption of macronutrients were tested, are described under each nutrient class herein. These studies do not particularly investigate the possible mechanism by which EE causes malabsorption (as discussed for each nutrient), but some attempt has been made to pin down probable pathways.

**Table 2:** Experimental evidence for macronutrient malabsorption in Brazilian children with biopsy confirmed environmental enteropathy.

Test group						Comparison group				
Reference	Test	*n*	Characteristics	Age (range)	Findings	*n*	Character	Age (range)	Findings	Additional information
Neto 1984 [46]	Carbohydrate loading test/Oral tolerance test: Lactose and sucrose	43	Children with chronic diarrhea as the predominant symptom and variable degree of protein calorie malnutrition as classified by Gomez’s criteria using Harvard growth chart as reference.	1–49 months	Lactose (21/43 children) and sucrose (7/23 children) malabsorption was observed, as indicated by the inability to raise glycaemic levels to >25% over basal.	No	No	No	No	There is no mention if the same children were tested for all three sugars and had biopsy. Did not check for associations between histopathological grading and malabsorption, or malabsorption and growth.
	Triglyceride load test, margarine	38			The mean increase in plasma triglyceride concentration was 33%.	22	Same as reference [17] (below)	6–60 months	The mean increase in plasma triglyceride concentration was 96%	
Jove 1983 [17]	Carbohydrate loading test/Oral tolerance test: Lactose and sucrose	33	Asymptomatic well-nourished children with giardiasis. Nutritional status was evaluated by Gomez’s criteria using Harvard growth chart as reference.	15–60 months	Flat response to lactose (17/30 children) and sucrose (2/28 children) loading tests.	No	No	No	No	Did not check for associations between histopathological grading and malabsorption, or malabsorption and growth.
	Triglyceride load test, margarine	33			The mean increase in plasma triglyceride concentration was 56%.	22	Well-nourished infants, no GI symptoms and 3 negative stool examinations for ova and parasites.	6–60 months	The mean increase in plasma triglyceride concentration was 96%.	

The dual sugar assay using lactulose (disaccharide) with mannitol/rhamnose (monosaccharide), to test intestinal permeability and passive absorption respectively, is widely used as a proxy diagnostic for EE [33]. A study examining the breath excretion of ^13^CO_2_ following an oral dose (2 g/kg) of stable isotope labeled ^13^C-sucrose breath test (SBT) in asymptomatic Australian Aboriginal children (*n* = 18, 95% CI of age 8–16 months, mean length-for-age *z*-score of −1.9), reported significantly lower (4 versus 6%) cumulative percent dose recovered in breath at 90 min (cPDR90) when compared to healthy non-Aboriginal controls (*n* = 7, age range 4–60 months, length-for-age *z*-score not given). A significant inverse correlation was noted between cPDR90 and lactulose rhamnose ratio (LRR) (*r* = 0.67, 95% CI: 0.42–0.82), in the Aboriginal children (with and without diarrhea), which suggests that *impaired intestinal epithelial integrity* could lead to sucrose maldigestion [34]. On the contrary, Zambian adults with biopsy confirmed EE who underwent an optimized SBT, showed similar ^13^CO_2_ excretion over the experimental duration in comparison to their healthy Scottish counterparts [35]. This could suggest adequate sucrase-isomaltase enzyme (SI) activity even in EE, either compensated by the unaffected intestine or an upregulation of activity as an adaptation to EE or a high sucrose diet. In support, transcriptomic studies suggest that the brush border expression of sucrase-isomaltase enzyme (SI) and sodium-glucose transporter-1 (SGLT-1) were upregulated, in duodenal biopsies of children with EE (from Pakistan) as compared to age-matched healthy North American controls [36]. However, the current protocol of SBT discussed herein lacks sensitivity to detect mild to moderate SI deficiency and may require modification (Table 1) for further use.

An earlier systematic review summarizes studies that have tested carbohydrate malabsorption in children with SAM [37]. The review reports reduced disaccharide and monosaccharide absorption, with a higher prevalence of lactose malabsorption. In the studies reported, a variety of methods to measure carbohydrate malabsorption were used, such as, carbohydrate loading tests, fecal reducing substances or acidic pH, and a few had disaccharidase levels measured in jejunal biopsies. The studies with jejunal biopsy lacked histopathological confirmation of EE, and therefore the link between EE and malabsorption was not established. The disadvantages of these methods are provided in Table 1, and they may not reflect the true deficit in digestion/absorption or compensation by the remaining gut that was not tested in the time duration of the protocol. Nevertheless, the above evidence suggests the possibility that the BBM enzyme lactase is more sensitive/vulnerable in an EE setting and may serve as an early or better marker of malabsorption. Therefore, lactose breath tests can be employed with the suggested modification in Table 1, to evaluate disaccharide malabsorption, in children with EE.

#### Proteins

##### Mechanisms with supporting evidence

The high prevalence of hypochlorhydria in malnourished children could theoretically lead to low pepsin activity and downstream consequences of SIBO [38, 39]. There is a great deal of redundant proteolytic activity in the small intestine, with the proportional contribution of pepsin to protein digestion of only ~10–20%. This has been experimentally demonstrated in adults who have undergone gastric bypass surgeries, in whom normal dietary milk protein digestion was reported [40]. On the contrary, reduced protein assimilation has been noted on gastric acid suppression therapy (with proton pump inhibitors for 2 days), but the quantitative contribution may be unimportant [39].

In addition to the consequences of mucosal damage other factors may be implicated in poor absorption. Elevated circulatory cortisol levels as observed in undernutrition with infection or inflammation may impair jejunal peptide transport as was experimentally observed in broilers receiving dexamethasone [7, 41]. In this stress induced (dexamethasone) dose-response study (administered at 0.1, 0.5, and 2.5 mg/kg body weight), the broilers had altered jejunal mucosal morphology akin to EE and showed reduction of glycylsarcosine (artificial dipeptide) transport in an everted jejunal sac experiment, for all three doses, suggesting lower peptide (PePT-1) transporter activity [41]. A direct inhibitory effect of the E-coli toxin LPS has also been observed on Na^+^-dependent AA transport (leucine), similar to its action on D-glucose transport [32]. On the contrary, rats infected by *Cryptosporidium parvum* [42], as in EE [43], show a compensatory post-translational upregulation of PePT-1 during acute infection, which maintained the ex-vivo glycylsarcosine transepithelial flux across the ileal mucosa in comparison to non-infected rats [42]. In summary, the extent of local (gut) or systemic infection or inflammatory state could cause protein malabsorption by directly or indirectly inhibiting BBM transporters, with the potential for compensatory upregulation. There is potential for tests using stable isotope AA tracers or glycylsarcosine (Table 1), to investigate these mechanisms in EE.

##### Evidence for protein malabsorption

There are very few studies in asymptomatic children with EE on the digestive or absorptive capacity of protein or AAs. A recent study conducted in children (aged between 18–24 months) from urban slums in South India, who were classified using a lactulose rhamnose ratio (LRR) cut-off, into EED (LRR ≥ 0.068) and no-EED (LRR < 0.068), showed no statistically significant difference between groups for the systemic availability (after digestion and absorption) of AAs from dietary protein. The dietary protein source tested in this study was an intrinsically labeled mung bean and uniformly labeled spirulina protein [44]. There was also no difference in true phenylalanine digestibility or its absorption index between these EED groups [44]. One possible reason for this observed indifference could be the functional adaptation of the gut epithelium, by either upregulation of PePT-1 transporters or proteases [42].

A study from the past, conducted in adults, point towards AA malabsorption in EE [45]. In healthy asymptomatic Indian men with histopathological features of EE on jejunal biopsy, the mean glycine (AA) and glycylglycine (peptide) absorption from the upper jejunum was lower by 31% when compared to age matched English men [45]. The mechanism for this finding was not investigated, but the researchers suggested theoretical possibilities as pointed out in the mechanisms common for all macronutrients section (above). Contrarily, the finding of lower AA absorption could otherwise imply the role of adaptation to higher protein intakes (mostly animal source) in the English men. Overall, there is some evidence in adults but not children, to indicate AA malabsorption, in EE. Studies using novel approaches using stable isotope AA tracers or glycylsarcosine (Table 1), to determine AA or peptide malabsorption, could be performed after standardization and validation, in children from different geographical settings, with varying degree of EE, preferably confirmed by biopsy.

#### Fats

##### Mechanism of fat malabsorption with supporting evidence

Dysbiosis is associated with deconjugation (removal of taurine or glycine) of bile salts (BS) to their constituent bile acids (BA), which could decrease the BS levels below the critical micellar concentration for fat absorption, thus causing fat malabsorption [17]. In the past, the triglyceride load test using margarine was used to assess fat malabsorption in children with biopsy confirmed EE (Table 2). As mentioned in Table 2, children with giardiasis or chronic diarrhea showed only half or one third increase in plasma triglyceride concentration when compared to a control group [17, 46]. The probable mechanistic pathway implicated for reduced fat absorption in these children was the presence of a higher rate of deconjugation of BS in their jejunal aspirates. The reported mean concentration of deconjugated and conjugated BA was 20.1 (SD 15.5) µmol/mL and 18.2 (SD 16.5) µmol/mL, respectively, in children with chronic diarrhea [46]. The level of deconjugated BA in the duodenal aspirates of normal adults (aged 18–45 years) is noted to be <1 µmol/mL [47]. Barring the considerable variation in collection of bile, introduced by time from meals, enterohepatic circulation, synthetic capacity of the liver, and transit time of intestinal contents, the concentration of deconjugated BA seems to be high in these children, suggesting the possibility of causing fat malabsorption.

The Study of Environmental Enteropathy and Malnutrition (SEEM) conducted in Pakistan observed a significant positive correlation (*r*_s_ = 0.32, 95% CI 0.064, 0.543) between percentage plasma glycocholic acid (a primary conjugated BA) and the total EED histopathological score of Pakistani children (*n* = 55, aged ~24 months) with EED, who were unresponsive to a 2–3 months nutritional intervention [48]. This finding probably indicates sub-clinical cholestasis, as proposed by the study researchers, which means lower concentration of BS in the intestine. Whereas, in another cross-sectional study conducted in Malawian children aged between 12–59 months, with suspected EE (a lactulose mannitol ratio cut-off of ≥0.15), the total median age adjusted serum BA were significantly lower in children with EE (4.51 versus 5.10 mM/L) compared to those without [49]. Additionally, the proportion of BAs conjugated with taurine instead of glycine was modestly but significantly higher in children with EE, in this study [49]. Both these findings indicate altered bile acid metabolism, but do not directly suggest the possibility of intraluminal deconjugation of BS by SIBO. Overall, this mechanism is not well supported by evidence, although it is likely to occur in children with EE, and therefore needs further investigation, using reliable tests to measure fat malabsorption (Table 1) and linking it to intraluminal (duodenal or jejunal aspirate) BS/BA concentration.

#### A summary, with gaps in research, and future directions

Multiple interlinking pathophysiological pathways leading to sub-optimal availability of macronutrients are implicated/proposed in EE, which could either act in tandem or in synchrony. There is some experimental evidence of lactose, AA and fat malabsorption in support of these proposed mechanisms. The potential for intestinal plasticity with the available reserve capacity may dampen the impact on growth, and lead to catch-up growth in late childhood or adolescence. On the contrary, the time taken for adaptation may be long enough to cause irreversible deficits in domains (immune system, brain) that have critical time windows for development. Future research should focus on understanding the degree of malabsorption of macronutrient, in different populations of children with EE, by adopting and standardizing available protocols. Where facilities permit, colonic biopsies can be conducted to determine if the large intestine is affected. Additionally, functional colonic contribution to the body’s nutrition economy could be explored. Longitudinal studies to establish causal links between EE related malabsorption and growth faltering are necessary, especially in high-risk settings, for early detection and prevention of deficits. Interventions with PERT, pre-digested fat/peptides could be explored in high prevalence areas to establish whether restoration of key nutrients is beneficial or not. In conclusion, the research gaps identified in this review, paves way for meaningful investigations and interventions in EE.
