# Correction: Is there dietary macronutrient malabsorption in children with environmental enteropathy?

**DOI:** 10.1038/s41430-024-01538-1

**Published:** 2024-11-07

**Authors:** Nirupama Shivakumar, Douglas J. Morrison, Shalini G. Hegde, Anura V. Kurpad, Paul Kelly

**Affiliations:** 1 Division of Nutrition, St. John’s Research Institute, St. John’s National Academy of Health Sciences (A Unit of CBCI Society for Medical Education), Bangalore, India; 2 Center for Doctoral Studies, Manipal Academy of Higher Education, Manipal, India; 3 Scottish Universities Environmental Research Centre (SUERC), University of Glasgow, Glasgow, UK; 4 Department of Pediatric Surgery, St. John’s Medical College Hospital, St. John’s National Academy of Health Sciences, Bangalore, India; 5 Department of Physiology, St. John’s Medical College, St. John’s National Academy of Health Sciences, Bangalore, India; 6 Blizard Institute, Barts and The London School of Medicine and Dentistry, Queen Mary University of London, London, UK; 7 Tropical Gastroenterology and Nutrition Group, University of Zambia School of Medicine, Lusaka, Zambia

**Keywords:** Metabolism, Pathogenesis

Correction to: *European Journal of Clinical Nutrition* 10.1038/s41430-024-01510-z, published online 08 October 2024

Affiliation 2 has been corrected from “Manipal Academy of Higher Education, Manipal, India” to “Center for Doctoral Studies, Manipal Academy of Higher Education, Manipal, India”. The original article has been corrected.

